# Enhancement of Biofunctionalization by Loading Manuka Oil on TiO_2_ Nanotubes

**DOI:** 10.3390/nano12030569

**Published:** 2022-02-07

**Authors:** Seo-Young Kim, Yu-Kyoung Kim, Yong-Seok Jang, Min-Ho Lee

**Affiliations:** Department of Dental Biomaterials, Institute of Biodegradable Material and Oral Bioscience, School of Dentistry, Jeonbuk National University, 567, Baekje-daero, Deokjin-gu, Jeonju-si 54896, Jeollabuk-do, Korea; mast6269@nate.com (S.-Y.K.); yk0830@naver.com (Y.-K.K.); yjang@jbnu.ac.kr (Y.-S.J.)

**Keywords:** Ti mesh, anodization, manuka oil, antibacterial activity, guided bone regeneration, biofunctionality

## Abstract

Metallic implants (mesh) for guided bone regeneration can result in foreign body reactions with surrounding tissues, infection, and inflammatory reactions caused by micro-organisms in the oral cavity after implantation. This study aimed to reduce the possibility of surgical failure caused by microbial infection by loading antibacterial manuka oil in a biocompatible nanostructure surface on Ti and to induce stable bone regeneration in the bone defect. The manuka oil from New Zealand consisted of a rich β-triketone chemotype, leptospermone, which showed strong inhibitory effects against several bacteria, even at very low oil concentrations. The TiO_2_ nanotubular layer formed by anodization effectively enhanced the surface hydrophilicity, bioactivity, and fast initial bone regeneration. A concentration of manuka oil in the range of 0.02% to less than 1% can have a synergistic effect on antibacterial activity and excellent biocompatibility. A manuka oil coating (especially with a concentration of 0.5%) on the TiO_2_ nanotube layer can be expected not only to prevent stenosis of the connective tissue around the mesh and inflammation by microbial infection but also to be effective in stable and rapid bone regeneration.

## 1. Introduction

In the clinical field of dentistry or orthopedic surgery, guided bone regeneration (GBR) using a mesh or membrane is a very important technique that has been applied in the augmentation of alveolar bone to local areas and the restoration of bone defects around implants [1,2]. Currently, titanium, a commercially available medical metal implant, is mainly used for this technique. It is known that titanium has good physical and mechanical properties compared to other biomaterials and has excellent biocompatibility; therefore, it is suitable for implants in the human body in the dental and medical fields [3,4]. In particular, it has been widely used in the treatment of maxillofacial fractures and reconstructions in the field of oral and maxillofacial surgery, and its efficiency and safety have been verified.

However, it has been reported that metallic implants have disadvantages, such as foreign body reactions with surrounding tissues, infection, and inflammatory reactions caused by micro-organisms in the oral cavity after implantation [5,6]. In recent research on GBR membranes, prevention of bacterial infection, wound healing, and induction of bone regeneration after GBR surgery were found to be very important factors for the success of the surgery [7]. Therefore, it was assumed that developing a biocompatible implant with antimicrobial properties against representative micro-organisms and general pathogens present in the oral cavity can increase the success rate of implantation in the dental or orthopedic clinical field. In order to stably use a material with antibacterial activity in the body, both biocompatibility and biosafety must be considered. Therefore, it is necessary to maximize the excellent biocompatibility on the surface of the Ti mesh while also increasing its biofunctionality by loading an antibacterial or anti-inflammatory material.

Among various antibacterial materials, antibacterial extracts derived from natural products provide relatively better biosafety for the human body than artificial antimicrobial agents. In addition, natural extracts have excellent flavor and antibacterial, antifungal, antimicrobial, bactericidal, and insecticidal properties [8,9]. Manuka essential oil derived from the East Cape region of New Zealand is extracted from the tea tree. It is composed of significant quantities of triketones and is known to exhibit excellent antibacterial properties [10]. However, its antibacterial properties have been proven only against some oral disease-related bacteria, and research on its effect on biocompatibility in combination with implants is insufficient. Therefore, through this study, we will attempt to verify the antibacterial effect of manuka oil against various bacteria-related oral diseases and opportunistic bacteria and to identify an effective coating concentration. We intend to develop a Ti mesh for functional bone regeneration treatment by applying manuka oil to the implant surface.

Recently, studies have been conducted on antibacterial coating agents by forming various shapes and components on the surface [11,12,13,14,15]. In particular, a nanostructure can increase the surface area in contact with agents and effectively load drugs on the implant’s surface. Therefore, it was thought that it would be useful to load natural antibacterial agents (manuka oil) after forming nanostructures on the surface of the Ti mesh for the development of biofunctional implants. In this respect, the methods of forming nanotube-type TiO_2_ on the titanium surface by anodization are expected to be sufficient for maximizing the efficiency of manuka oil loading. Moreover, since TiO_2_ nanotubes effectively promote the proliferation and differentiation of osteoblasts [16,17], they can be applied to GBR meshes used to treat bone defects.

This study aimed to reduce the possibility of surgical failure caused by microbial infection of Ti mesh by loading manuka oil after biocompatible pre-surface treatment and inducing stable bone regeneration in the bone defect. First, the antibacterial activity against various bacteria and cytocompatibility of osteoblasts was evaluated to prove the effectiveness of the concentration of manuka oil. After coating the anodized surface of the Ti mesh with the optimal concentration of manuka oil with high antibacterial activity and low cytotoxicity, the effect on regeneration of the bone defect under the mesh was evaluated.

## 2. Materials and Methods

### 2.1. Material Preparation

Pure titanium with a 0.1 mm thickness (Kobe Steel Ltd., Kobe, Japan) was cut into squares (10 × 10 mm). The specimens were immersed in an etching solution of 7 wt.% HF and 12 wt.% HNO_3_ in distilled water (DW). After washing the specimens gently in DW, they were dried in an oven at 40 °C for 1 d and subsequently subjected to surface treatments.

As the carrier, the surface of Ti was anodized using a DC constant power supply (Daunanotek Co., Ltd., Gwangmyeong-si, Korea). The anode and cathode of device were connected as Ti specimen and platinum plate, respectively. Anodization was performed at 20 V for 1 h in an electrolyte with NH_4_F (1 wt.%) and glycerol (79 wt.%). The specimens were then heated at 5 °C/min to 500 °C in a high-temperature heating oven (US/CC59256, A Jeon Heating Industrial Co., Namyangju-si, Korea). The temperature was held at 500 °C for 2 h and then cooled to 25 °C in a furnace.

Manuka essential oil (Manuka Natural Ltd., Christchurch, New Zealand) was used as an antibacterial coating material to improve the biofunctionality of the implant. The oil made from steam-distilled leaves in the East Cape region of New Zealand. The chemical bonding structure of this oil was confirmed by Fourier transform infrared (FT-IR) spectroscopy (Spectrum GX, Perkin Elmer Co., Norwalk, CT, USA) in the IR range of 500–4000 cm^−1^. Based on the antibacterial test results, 0.1%, 0.5%, 1%, and 2% manuka oil were selected as coating concentrations. The manuka oil was prepared by dilution with mineral oil (Bio-Rad Laboratories, Hercules, CA, USA). Ten μL of different concentrations of manuka oil were dropped onto each Ti nanotube surface (1 cm^2^) to estimate their effects on the Ti nanotubes. The surface was then completely dried in a vacuum oven at 37 °C. Table 1 lists the conditions of surface treatment for each group.

### 2.2. Antibacterial Activity of Manuka Oil

#### 2.2.1. Preparation of Bacterial Strains

*Streptococcus mutans* KCTC3065, *Streptococcus anginosus* KCTC3983, *Escherichia coli* KCTC1682, *Staphylococcus epidermidis* KCTC1917, and *Porphyromonas gingivalis* KCTC5352 were chosen as the test bacterial strains. All bacterial strains were procured from the Korean Collection for Type Cultures (KCTC) and pre-cultured on a sheep-blood agar plate (BAP; ASAN Pharmaceutical, Seoul, Korea) for bacterial growth assays. *P. gingivalis* was cultured by maintaining for one week at 37 °C under anaerobic conditions using the GENbox jar (96127; BioMérieux SA, Marcy-l’Etoile, France) with the GENbox anaer system (96124; BioMérieux SA, Marcy-l’Etoile, France), and the rest of the strains were grown in an incubator for 48 h at 37 °C in the presence of 5% CO_2_. A standardized suspension of all bacterial strains was diluted with brain–heart infusion (BHI; Difco, Becton Dickinson and Company, Sparks, MD, USA) broth to contain a bacterial count of 1.5 × 10^7^ colony-forming units (CFU)/mL.

#### 2.2.2. Determination of Minimum Inhibitory Concentration (MIC) and Minimum Bactericidal Concentration (MBC)

MIC and MBC determinations were used to assess the antimicrobial efficacy of manuka oil against all bacterial strains. To determine the most potent antibacterial component, the agent (manuka oil) ranged from 10% to 0.019% using the broth microdilution method described in ISO 20776-1:2019 [18], and then the MIC was determined. A standardized suspension (100 μL) of each strain was added to 100 μL of the diluted agents. After incubation in a 5% CO_2_ incubator at 37 °C for 24 h (for one week under anaerobic conditions for *P. gingivalis*), absorbance was measured using an enzyme-linked immunosorbent assay (ELISA) microplate reader (EMax^®^ Precision Microplate Reader; Molecular Devices, Sunnyvale, CA, USA) at a wavelength of 600 nm. Then, the differences in absorbance values before and after incubation were compared. MIC was determined as the lowest concentration that inhibited bacterial growth, with an absorbance gap lower than 0.01 at 600 nm.

An aliquot (100 μL) was extracted in groups with concentrations higher than the MIC. To determine MBC, the aliquot was spread on the surface of BAP and subcultured. After incubation, including the MIC test, MBC was determined as the lowest concentration without visible bacterial growth on BAP.

#### 2.2.3. Antimicrobial Sensitivity Test by Disc Diffusion Assay

Sensitivity and resistance of the bacteria to manuka oil were determined using the disc diffusion method. Sterile paper discs (156100; BIOZOA Biological Supply Company, Seoul, Korea) of 6 mm diameter were used in this assay. The discs were impregnated with 100 μL of diluted manuka oil and dried. A blank disc soaked in only BHI was used as a negative control. All strains were inoculated into the BAP by streaking a sterile cotton swab dipped in a standardized bacterial suspension. The prepared discs were positioned on the surface of the inoculated agar plates. After incubation, including the MIC test, the diameter of the inhibition zone around the disc (including the diameter of the disc) was measured.

### 2.3. Surface Analysis

After surface treatment with different concentrations of manuka oil, changes in morphology and compound were observed using a field emission scanning electron microscope (FE-SEM; SUPRA40VP, Carl Zeiss Co., Oberkochen, Germany) with energy-dispersive spectroscopy (EDS) capabilities. Analysis of the crystal phase of the surface was performed in the 2θ range, from 10° to 90°, using a multipurpose high-performance X-ray diffractometer (XRD; X’PERT-PRO Powder; PANalytical Co., Almelo, The Netherlands) installed at the Center for University-Wide Research Facilities (CURF) at Jeonbuk National University. XRD analysis was carried out under a voltage-current condition of 40 kV and 30 mA by Cu-Kα radiation (λ = 0.154060 nm) at a scanning rate of 0.03° s^−1^. Changes in the chemical bonding structure on the surface were confirmed by FT-IR at a resolution of 4 cm^−1^ and in a range of 500–4000 cm^−^^1^.

Wettability of the surface with different coating concentrations was tested by measuring the contact angle. A biological Hanks’ balanced salt solution (HBSS; H2387; Sigma-Aldrich, St. Louis, MO, USA) [19] was determined as testing liquid for this experiment. It is water-soluble simulated body fluid (SBF). It was automatically dropped with same volume onto each surface using the touch-drop method in a contact-angle analyzer (Phoenix-300 Touch; Surface Electro Optics, Suwon-si, Korea). The test was progressed in the air, and images of the dynamic contact angle were obtained by sequentially capturing 40 frames per 300 ms interval. The third frame captured after HBSS was touched on the surface was compared between each group.

### 2.4. Immersion Test

For bioactivity evaluation, the specimens sterilized by ethylene oxide gas were placed in a 5% CO_2_ incubator at 37 °C for five and ten days after immersion in SBF adjusted to a pH of 7.4. The SBF was replaced every two days to maintain its concentration. After immersion, characterization of the surface was performed using FE-SEM with EDS and XRD. For the observation of FE-SEM, Pt was sputtered to provide conductivity on the surface of each group.

### 2.5. Cytotoxicity Test

Cytocompatibility on the surface and manuka oil was examined using mouse osteoblast cells (MC3T3-E1) received from the American Type Culture Collection (Manassas, VA, USA). α -minimum essential medium (α-MEM; Gibco Co., Grand Island, NY, USA) containing 10% fetal bovine serum (FBS; Gibco Co., Grand Island, NY, USA), 500 U/mL penicillin (Gibco Co., Grand Island, NY, USA), and 500 U/mL streptomycin (Gibco Co., Grand Island, NY, USA) was used as the culture medium. An initial density (2.0 × 10^4^ cell/mL) of cell solution was fabricated using the pre-cultured cells for seeding cells. A cytotoxicity test for this study was classified with two cases (direct and indirect cytotoxicity test, with specimens (oil on the surface) or without specimens (only oil), respectively).

One case was prepared by seeding cells directly on specimens loaded with different concentrations of manuka oil (10 μL) in blank 24-well plates. Another case was prepared by dividing the blank 48-well plates and then adding the medium with different concentrations of manuka oil (10 μL). After cells were cultured, their viability and differentiation were measured using assay kits. For the direct cytotoxicity test, the cells were cultured for five days at 37 °C in an incubator containing 5% CO_2_ and analyzed using a water-soluble tetrazolium salt (WST) assay and osmium staining. For the indirect cytotoxicity test, the cells were cultured for 2, 4, 7, and 21 days for analysis by 3-(4,5-dimethylthiazol-2-yl)-2,5-diphenyltetrazolium bromide (MTT), alkaline phosphatase (ALP), and alizarin red S (ARS) assays, as well as crystal violet staining.

#### 2.5.1. Cell Proliferation Analysis

- WST assay

A cytotoxicity test was conducted using the Cell Counting Kit-8 (Enzo Life Sciences Inc., NY, USA). The culture medium was replaced with water-soluble tetrazolium salt (WST)-8 reagent [2-(2-methoxy-4-nitrophenyl)-3-(4-nitrophenyl)-5-(2,4-disulfophenyl)-2H-tetrazolium, monosodium salt]. Then, the cell plate with WST-8 reagent was kept in an incubator containing 5% CO_2_ for 1.5 h at 37 °C. The absorbance of a water-soluble formazan dye produced by the reagent was measured at 450 nm using an ELISA microplate reader.

- MTT assay

The dyeing solution for analysis of proliferation was prepared by mixing 3-(4,5-dimethylthiazol-2-yl)-2,5-diphenyltetrazolium bromide (MTT; Sigma Chemical Co., St. Louis, MO, USA) solution with a 10% ratio to α-MEM. The culture medium was replaced with the MTT solution. After incubation for 4 h, the solution was removed. To dissolve the dark-blue crystals of MTT formazan, dimethyl sulfoxide (DMSO; Duksan Pure Chemical Co., Ltd., Ansan-si, Korea) was added to each specimen. Absorbance after solubilization of the formazan was measured at a wavelength of 540 nm using an ELISA microplate reader.

#### 2.5.2. Cell Morphological Analysis

- Osmium staining

For cell staining after culturing for five days, the cells were fixed in 25% glutaraldehyde for 2 h, then washed with phosphate-buffered saline (PBS). For the secondary fixing step, cells on the specimen were treated for 2 h in a 1% osmium tetroxide solution. The solution was removed, and specimens were then washed with PBS. Afterward, the cells were dehydrated by immersion for 10 min each in 30%, 50%, 70%, 80%, 90%, 95%, and 100% ethanol. The morphology of cells on the surface-treated specimens was observed by scanning electron microscopy (SEM; JSM-5900, JEOL, Tokyo, Japan).

- Crystal violet staining

After culturing for two and four days, the culture media were removed and washed with PBS. The cells were fixed with a mixture of 0.2% glutaraldehyde and 3% formaldehyde, washed with PBS, and stained with 0.3% crystal violet. The morphology of cells was examined using an optical microscope (DM2500M; Leica, Wetzlar, Germany).

#### 2.5.3. Cell Differentiation Analysis

The differentiation of cells after culturing for seven and fourteen days was analyzed by an alkaline phosphatase (ALP) assay kit (MK301, TaKaRa Bio, Shiga, Japan). To compare the expression of ALP activity in each group, the cultured cells were lysed with 1% NP-40 based on the saline. Then, a mixture containing p-nitrophenyl phosphate was added and mixed. After incubation for 30 min at 37 °C, absorbance was measured at 405 nm using an ELISA microplate reader.

#### 2.5.4. Quantification of Mineralization Analysis

After culturing for 21 days, the cultured cells were washed three times with saline and fixed with 4% formaldehyde for 15 min. After removing the fixation solution and rewashing, cells were stained with 40 mM alizarin red S (ScienCell Research Laboratories, Inc., Carlsbad, CA, USA) solution and washed after 20 min. The ARS-stained cells were treated for 15 min with 10% cetylpyridinium chloride (Sigma-Aldrich Chemical Co., St. Louis, MO, USA) solution prepared in 10 mM trisodium phosphate to quantify calcium in cells. The absorbance of the extracted solution was measured at 560 nm using an ELISA microplate reader.

### 2.6. In Vivo Test

**Figure 1:**
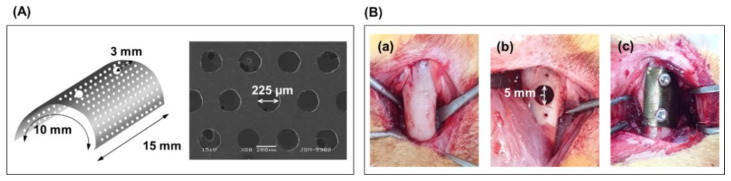
Implantation of the surface-modified Ti mesh on the femoral defect for four and six weeks; (**A**) Design of Ti mesh and (**B**) procedure of surgery [(**a**) femur in rabbit, (**b**) Formation of defect with a diameter of 5 mm in femur, (**c**) Fixation of the surface-modified Ti mesh on the femoral defect].

The current study was conducted in compliance with the Declaration of Helsinki and was approved by the Institutional Animal Care and Use Committee of the Jeonbuk National University Laboratory Animal Center (CBNU 2018-054). Based on previous experimental results, the T, TN, and TN0.5M groups were selected as the experimental groups, and the group forming only defects was used as the control group. Twenty-four square specimens were cut to a size of 10 × 15 × 0.1 mm^3^ using a Ti mesh (TMN35508-S1, Neo Biotech, Seoul, Korea) with 225 μm diameter holes (Figure 1A) to induce GBR of bone defects. Four specimens were prepared for each experimental group. The specimens were placed on both femurs of rabbits. 

Sixteen male white New Zealand rabbits (eight weeks old) weighing approximately 1.8–2 kg were used for this study. The rabbits were anesthetized via intramuscular injection of 0.7 mL/kg of ketamine hydrochloride (Ketalar, Yuhan, Seoul, Korea) and 0.2 mL/kg of xylazine hydrochloride (Rompun, Bayer Korea, Seoul, Korea). The surgical site was disinfected with povidone–iodine, and infiltration anesthesia was induced by injecting 2% lidocaine (lidocaine-HCl, Huons, Seoul, Korea). Skin, fascia, and periosteal incisions were performed by surgically exposing the distal femoral bone. The position of the implant was determined to be 1 cm above the knee joint on the femoral side.

Through the cortical bone at the position, a circular bone defect with an 8-mm depth was formed using a trephine bur with a diameter of 5 mm (227 B, Komet, Germany). The defect was covered with a prepared Ti mesh. Then, the edge of the mesh was fixed using a Ti screw, as shown in Figure 1B(a). The soft tissue was sutured using 4–0 synthetic absorbable multifilament suture materials (VicrylPlus Antibacterial, Ethicon, Somerville, NJ, USA). Postoperative antibiotics (0.15 mL/kg) were administered subcutaneously (Amikacin, Samu Median Co., Ltd., Yesan-Gun, Korea) at 0, 24, and 48 h after the operation. Eight rabbits were sacrificed at four and six weeks after implantation, and Ti mesh-guided femoral blocks were obtained.

#### 2.6.1. Histological Observation

After implantation of the surface-modified Ti mesh for four and six weeks, histological analysis was conducted for observation of the bone regeneration in the bone defect covered by the surface of mesh. To stain the bone tissue, the mesh-implanted femoral part fixed in a 10% formalin solution was stained in Villanueva Osteochrome Bone Staining solution (Polysciences, Inc., Warrington, PA, USA) for 3 days. For dehydration of bone, the stained bone was immersed in increasing concentrations of ethanol (from 80% to 100%). It was then embedded by polymerization in methyl methacrylate (Yakuri Pure Chemical Co., Kyoto, Japan) containing benzoyl peroxide (Sigma-Aldrich, St. Louis, MO, USA). The embedded blocks were sectioned and examined using an optical microscope.

#### 2.6.2. Bone Regeneration Analysis by Micro-CT

To evaluate the shape, bone volume (BV), and bone mineral density (BMD) of new bone formed inside the defect area after the in vivo study, the bone, including the screws, was scanned using microcomputed tomography (Micro-CT; Skyscan 1076, Skyscan, Belgium). The 3D images were reconstructed using Bruker Skyscan free software (DataViewer, CTAn, and CTvox).

### 2.7. Statistical Analysis

Statistical analysis of the results in this study was conducted using one-way analysis of variance with Tukey tests. When the *p*-value was lower than 0.05, the difference between the groups was considered statistically significant.

## 3. Results

### 3.1. Antibacterial Activity of Manuka Oil

The antimicrobial activity of manuka oil was evaluated against *Streptococcus mutans*, *Streptococcus anginosus*, *Staphylococcus epidermidis*, *Porphyromonas gingivalis*, and *Escherichia coli*. As shown in Table 2, the MIC results of manuka oil against *S. mutans*, *S. anginosus*, *S. epidermidis*, *P. gingivalis*, and *E. coli* were 0.08%, 0.08%, 0.02%, 0.16%, and 1.25%, respectively, with MBC results of 2.5%, 0.31%, 0.31%, 1.25%, and 10.0%, respectively. Manuka oil showed inhibitory activity against all strains. In particular, a better antibacterial effect was observed against Gram-positive bacteria than against Gram-negative bacteria.

**Figure 2:**
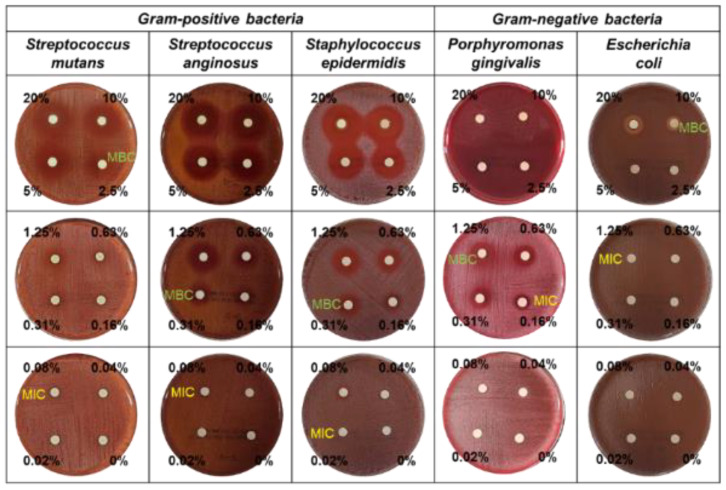
Antibacterial activity by disc diffusion test with the different concentrations (0.02–20%) of manuka oil against the five bacterial species.

Disc diffusion tests confirmed the inhibitory effect of manuka oil against all tested strains (Table 2 and Figure 2). The diameter of the inhibition zone against *S. mutans* expanded from 1.3 mm to 12.4 mm when manuka oil concentration was increased from 0.31% to 20%. The diameter of the inhibition zone against *S. anginosus* increased from 1.4 mm to 12.3 mm with manuka oil concentrations over 0.08%; against *S. epidermidis* from 0.6 mm to 10.9 mm with manuka oil concentrations over 0.04%; and against *P. gingivalis* from 1.5 mm to over 2.5 mm with manuka oil concentrations over 0.08%. The inhibition zone against *E. coli* was observed with manuka oil concentrations over 5%, and its diameter was narrow, even at high concentrations of manuka oil.

Among all the tested strains, the inhibition sensitivity at low concentrations of manuka oil was highest against *S. epidermidis*, while the strength of inhibitory activity was strongest against *P. gingivalis*.

### 3.2. Surface Characterization of Ti Modified by Anodization and Manuka Oil

**Figure 3:**
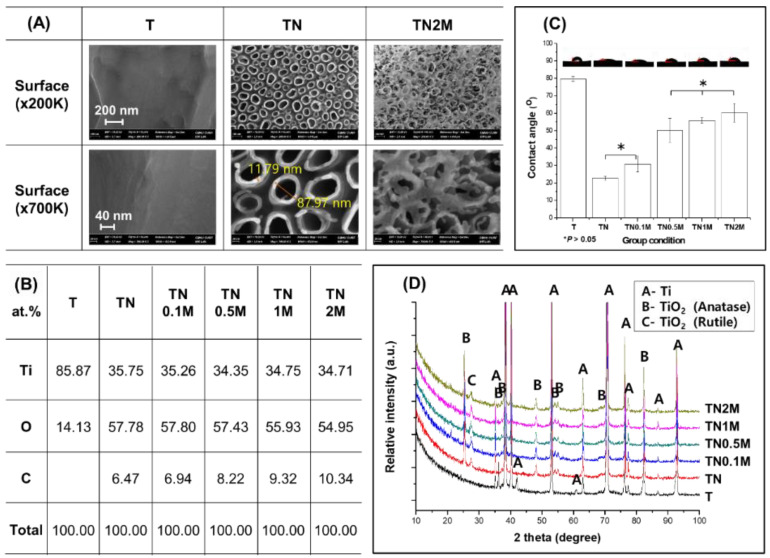
Change in surface properties before and after formation of nanotubes and coating with manuka oil. (**A**) FE-SEM images; (**B**) EDS analysis (at %); (**C**) contact angle value and images for comparison of surface wettability; (**D**) XRD patterns for analysis of phase transformations on the surface. * No significant difference (*p* > 0.05).

Anodization treatment led to the formation of self-aligned nanotubes on the Ti surface with a thickness of 11.9 ± 2.4 nm, a diameter of 88.3 ± 4.8 nm, and a height of about 600 nm, as well as increased concentration of O ions (Figure 3A,B). The C content on the surface increased with the coating concentration of manuka oil. In the TN2M group coated with the highest concentration of manuka oil, it was found that a translucent material covered the nanotubular surface, and the morphology of the nanotubes was still maintained.

It was confirmed that the TiO_2_ crystal structure (Figure 3D), which consists of anatase and rutile phases, was precipitated on the surface after anodization. The manuka coating did not affect the phases formed on the surface after anodization.

The surface contact angle of the TN group decreased significantly ((Figure 3C). However, a significantly increased contact angle was observed in the manuka-coated groups at concentrations greater than 0.5% in comparison to the TN group. Among the manuka-coated groups, the contact angle increased with the concentration of manuka oil, but all groups showed significantly lower surface contact angles than the untreated group (T).

**Figure 4:**
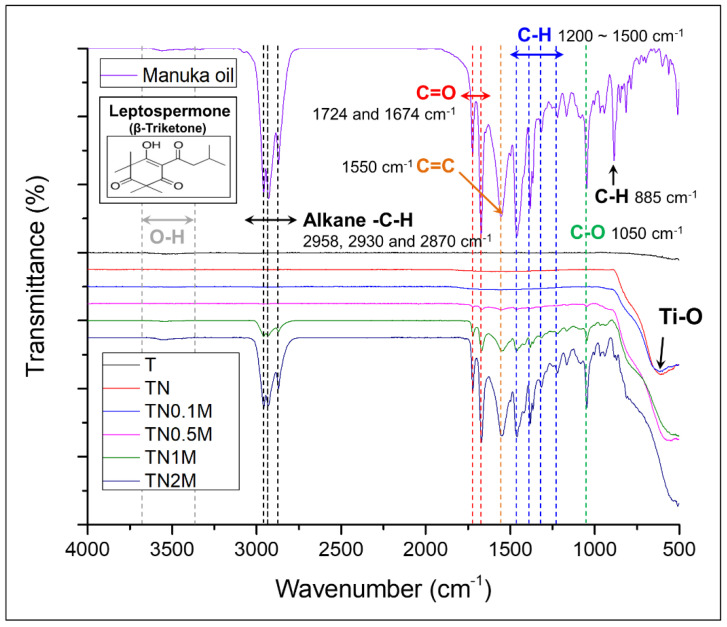
Chemical bonding analysis by FT-IR spectra. Manuka oil from the East Cape region of New Zealand/Ti surface before and after coating with anodization and different concentrations of manuka oil.

The FT-IR spectra of manuka oil (Figure 4) showed specific bands related to the aliphatic alkane (C-H) stretching vibrations (at 2958, 2930, and 2870 cm^−1^), strong aliphatic and conjugated ketone (C=O) stretching bands (at 1724 and 1674 cm^−1^), a medium cyclic alkene (C=C) stretching band (at 1550 cm^−1^), and a strong primary alcohol (C-O) stretching band (at 1050 cm^−1^).

After surface treatment by anodization, strong absorption bands were observed at 662 and 704 cm^−^^1^, owing to Ti–O vibrations in the TiO_2_ crystal structure. After coating the TiO_2_ surface with manuka oil, the same peaks exhibited in the FT-IR analysis of manuka oil were observed in the groups coated with a concentration of manuka oil greater than 0.5%. The intensity of the peaks became stronger with an increase in the manuka-coating concentration.

### 3.3. Bioactivity of Ti Modified by Anodization and Manuka Oil by Immersion Test in SBF

**Figure 5:**
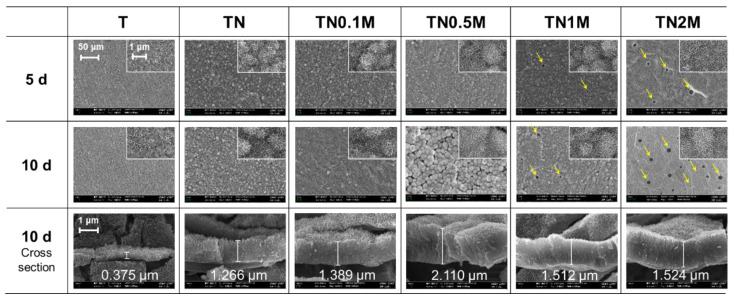
Morphological changes in the surface after immersion in SBF for five and ten days.

As a result of the SBF immersion test (Figure 5 and Table 3), Ca and P ions were detected on the surface of all groups, and the morphology of the surface changed to a flower-like apatite. 

Concentrations of Ca and P ions were significantly higher in the anodized group than in the untreated group. In addition, the apatite morphology became more stereoscopic. Cross-sectional images confirmed that apatite with a significantly thicker layer was precipitated on the anodized group than on the untreated group.

All manuka-coated groups showed a superior Ca and P precipitation capacity compared to the T and TN groups. The Ca/P ratio increased with the coating concentration of manuka oil. However, in the groups coated with manuka oil with a concentration over 1%, the bioactive layer was formed only on the rest of the surface, with the exception of the agglomerated area of the manuka oil. Thus, pores appeared to have formed on the surface (yellow arrows). As the concentration increased, the size of the pores increased.

**Figure 6:**
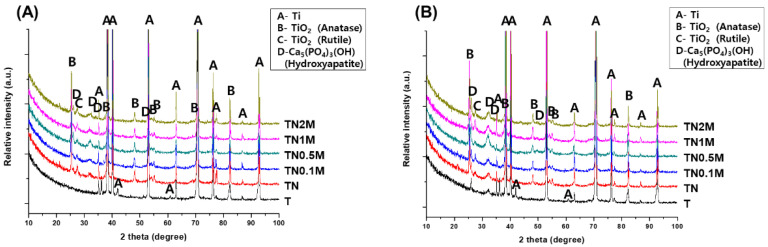
XRD patterns of the surface after immersion in SBF for (**A**) five days and (**B**) ten days.

After five days of immersion, the TiO_2_ peak formed by anodization was still detected in all surface-treated groups, except for the untreated group (Figure 6). In addition, the hydroxyapatite (HA) peak, which is a calcium phosphate compound, was widely identified at 32.054°. The intensity of the detected HA peaks was similar in each treatment group.

After ten days of immersion, the surface of the untreated group had new small HA peaks at 25.879° and 32.054°. On the surface of all treated groups, the intensity of the HA peaks that initially formed on the fifth day increased, and new HA peaks were additionally observed at 25.879° and 34.196°. In particular, all manuka-coated groups showed higher HA peak intensities than the untreated group.

### 3.4. In Vitro Cytotoxicity Test

**Figure 7:**
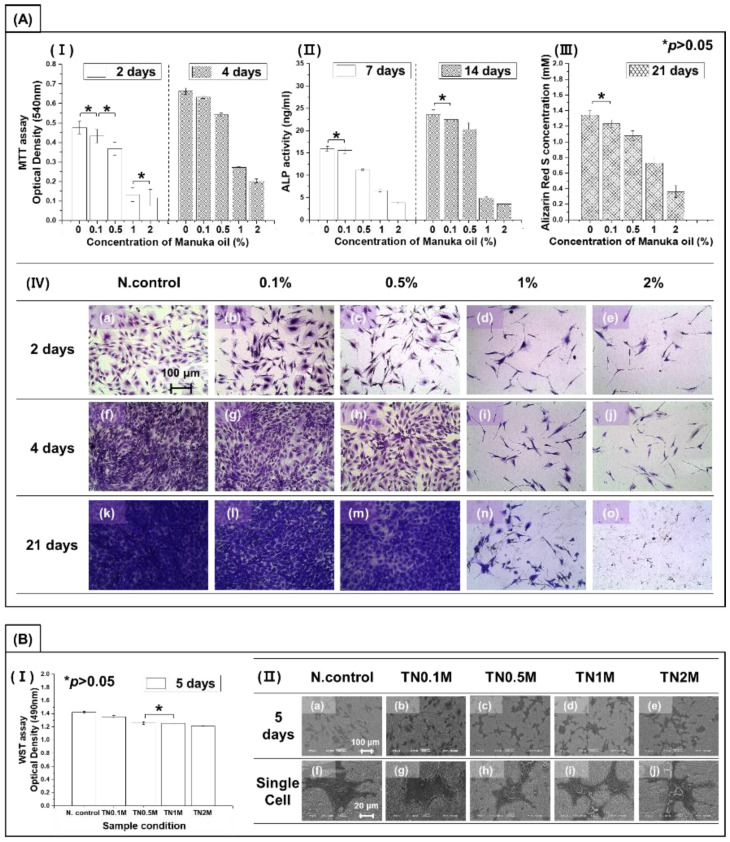
(**A**) Cytotoxicity test of MC3T3-E1 osteoblast cells in the medium containing manuka oil: (**I**) proliferation (by MTT assay); (**II**) differentiation (by ALP assay); (**III**) quantification of calcium deposits (by ARS assay); and (**IV**) morphology of cells (by crystal violet staining). (**B**) Cell viability test on surfaces coated with different concentrations of manuka oil: (**I**) proliferation (by WST assay); and (**II**) morphology of cells (by osmium staining). * No significant difference (*p* > 0.05).

Figure 7A shows the evaluation results of the viability of osteoblasts according to the coating concentration of manuka oil by calculating the actual amount of manuka oil contained on the manuka-coated surface. As a result of culturing for four days in a culture medium containing different concentrations of manuka oil, cell proliferation gradually reduced with increasing concentration. In particular, a surface-coating concentration of 1% or more showed a significant decrease of 50% or more in cell proliferation and differentiation ability compared to the negative control group (*p* < 0.05). In addition, the cytoplasmic extension of cells was reduced. Figure 7A(III) shows the evaluation results of calcified bone-cell mineralization by alizarin red S staining after culturing osteoblasts for 21 days. A surface-coating concentration of 0.5% or more showed significantly reduced mineralization compared to the negative control group (*p* < 0.05). In addition, this decrease was dependent on an increase in concentration. The number of visible cells in Figure 7A(IV) decreased, corresponding to the result in Figure 7A(I,III). The morphology of single cells was sharply spread with increasing concentration of manuka oil.

Figure 7B shows the results of analyzing the effect of the surface on cell activity through the direct cell culture method after coating the anodized surface with manuka oil. After five days of culture, the tendency for cell proliferation and differentiation was the same as when only oil was analyzed. However, it was found that the decrease in cell proliferation and differentiation with increased concentrations of manuka oil was significantly less when oil was combined with the anodized surface than when it was not. This confirms that coating nanotubes with manuka oil could mitigate the cytotoxicity of manuka oil and improve the cell activity of osteoblasts. In the cell morphological analysis, osteoblasts on the treated surface were grown with good cell adhesion. The cytoplasmic extension of cells decreased with the concentration of manuka oil, while cell–cell interactions showed a relatively increased pattern. The filopodia of cells were actively spread in all groups.

### 3.5. In Vivo Test

**Figure 8:**
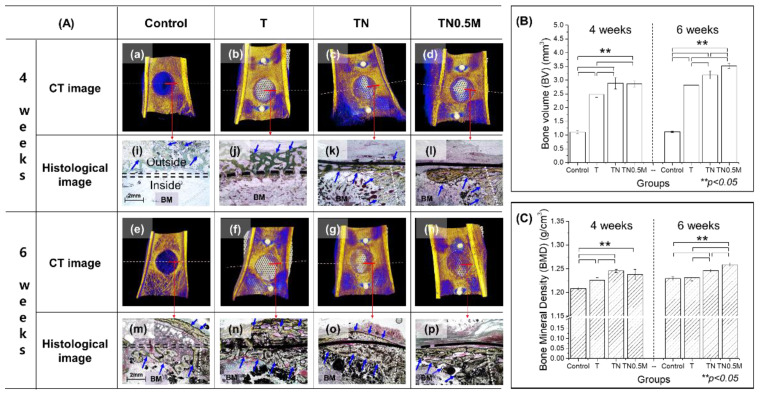
Bone regeneration inside the femoral defect after implantation of surface-modified Ti mesh for four and six weeks. (**A**) 3D images (**a**–**h**) and histological images (**i**–**p**) (new bone marked with blue arrows; BM means bone marrow); (**B**) bone volume (BV); (**C**) bone mineral density (BMD), ** significant difference (*p* < 0.05).

After four weeks of implantation, the callus, a type of soft bone, rapidly formed with a very low BMD value in the control group without mesh implantation (Figure 8A). The callus was excessively and widely formed in the upper part of the defect, even though its volume was very low (Figure 8B). In addition, even after eight weeks, the immature bone was still slowly remodeled into mature bone.

On the other hand, the group implanted with the untreated Ti mesh showed a mature bone formation with a significantly higher BMD value (Figure 8C) than that of the control group (*p* < 0.05). However, the position of bone formation in the reparative stage was overgrown on the upper part of the mesh, not inside the bone defect. The new bone formed in the TN group was thin but rapidly covered most of the bone defects. In the case of the TN0.5M group, new bone growth was focused on the edge of the bone defect in the early implantation stage, with a BV that was also lower than that in the TN group, but with no significant difference (*p* > 0.05). In addition, the new bone in this group was formed only in the lower part of the mesh, unlike in other groups, and it was thick.

After six weeks of implantation, the TN and TN0.5M groups showed superior bone-healing ability with significantly improved BMD values compared to the control group. As the TN group rapidly induced adhesive bone formation with the mesh, the bone actively and densely grew inside the bone defect, with a simultaneous increase in bone formation on the upper part of the mesh. The TN0.5M group showed enhanced bone formation with significantly higher BV and BMD values compared to the untreated and TN groups (*p* < 0.05). Moreover, TN0.5M group also exhibited almost perfectly completed bone regeneration of the bone defect, forming bone only inside the bone defect and not on the upper part of the mesh.

## 4. Discussion

In this study, TiO_2_ nanotubes with high bioactivity and manuka oil with an antibacterial effect were applied to the surface of Ti mesh to improve the biofunctionality of the titanium surface. First, a stable oxide layer was formed to impart bioactivity on the surface, and the surface morphology was modified to increase the contact area with manuka oil. As a result, self-aligned nanotubes with a constant thickness and height were formed on the Ti surface through anodization. These nanotubes were stable TiO_2_ oxide layers consisting of anatase and rutile structures. This oxide layer contributed to the formation of a super-hydrophilic surface with an increase in the surface area. The additional manuka oil coating did not affect the crystal structure or morphological changes in the TiO_2_ nanotubes. This confirms that the hydrophilic surface of the TiO_2_ nanotubular layer changed to a hydrophobic surface with increasing concentrations of manuka coating. However, owing to the super-hydrophilic surface of the nanotubular layer, the surfaces of all the manuka-coated groups were able to obtain a relatively improved wettability compared to that of the untreated Ti group in contact with the SBF.

Manuka oil is classified into three chemotypes: monoterpene-rich, sesquiterpene-rich, and triketone-enriched, according to the content of major components [20]. The triketone-enriched chemotype of manuka oil obtained from the East Cape region is rich in β-triketones. β-Triketones consist of flavesone, leptospermone, and iso-leptospermone [10]. FT-IR analysis confirmed that the manuka oil from New Zealand used in this study was composed of a leptospermone component with aliphatic C-H stretching vibrations and particularly strong carbonyl stretching bands. In addition, the manuka oil used was of a chemotype containing high triketones, and leptospermone was predominant among the β-triketone chemotypes. Even after applying manuka oil on the TiO_2_ surface, its active ingredients and chemical structures did not change. It was proven that the amount of β-triketone leptospermone per unit of area could be effectively increased as the peak intensity was enhanced, depending on the concentration of manuka oil.

In general, it has been reported that the presence of rich β-triketones offers a high level of antimicrobial activity against Gram-positive organisms, such as *Staphylococcus* spp., *Enterococcus* spp., and *Streptococcus* spp., as well as certain dermatophytic fungi [21,22]. In addition, the leptospermone of the β-triketone chemotype isolated from *L. scoparium* is known to exhibit unique and strong antimicrobial properties [23]. Therefore, in this study, proof of the effectiveness on the antibacterial effect of manuka oil containing β-Triketone leptospermone was obtained by various antibacterial tests against oral diseases and pathogenic organisms, such as *S. mutans, S. anginosus, S. epidermidis, P. gingivalis,* and *E. coli*. This study demonstrate that a considerable amount of leptospermone structure in manuka oil inhibited growth against all test strains, depending on the oil concentration. The effect of manuka oil against each strain was in the order of *S. epidermidis > S. anginosus > S. mutans > P. gingivalis > E. coli*, with MIC values of 0.02%, 0.08%, 0.08%, 0.16%, and 1.25%, respectively.

The manuka oil used in this study showed excellent antibacterial effects against Gram-negative and Gram-positive organisms. In particular, it was confirmed that manuka oil has significant antibacterial activity at a very low concentration of 0.2% or more in common against most bacteria, except *E. coli*. This is meaningful for antibacterial effect because manuka oil MIC values against some bacterial strains were similar or lower compared to that of propolis [24] and cinnamomum [25], which are representative essential oils extracted from natural products. Leptospermone highly loaded on the surface with an increase in the coating concentration can act effectively for antibacterial and sterilization on the surface of Ti mesh, since it showed a great inhibiting effect on bacterial growth as the concentration increased. Moreover, a wide expansion in the inhibition zone, depending on the oil concentration, was observed against *S. mutans* and *P. gingivalis*, which are representative oral disease-related bacteria. Thus, the application of manuka oil to dental implants can have a significant preventive and therapeutic effect against oral diseases.

However, since the goal of this study was to provide biofunctionality to the Ti mesh for GBR, it is necessary to confirm the appropriate concentration that does not inhibit bone formation while exhibiting excellent antibacterial properties. Therefore, the coating concentrations of manuka oil for the anodized Ti mesh, considering the antibacterial test results, were selected as 0.1%, 0.5%, 1%, and 2%, and their biocompatibility was evaluated.

Changes in the morphology, composition, and crystal phase on the surface of the Ti mesh were analyzed by immersing each surface-treated group in the SBF to evaluate the effects of TiO_2_ oxide layer formation and manuka oil coating on bioactivity. The porous TiO_2_ oxide layer formed on the surface after anodization showed super-hydrophilicity and could induce the formation of Ti-OH groups on the surface by a friendly reaction with SBF. The porous TiO_2_ oxide layer can therefore promote the formation of apatite nucleation through active binding with Ca^2+^ and PO_4_^2−^ ions in the SBF [19]. In this study, hydroxyapatite (HA), a calcium-phosphate compound, was rapidly precipitated and crystallized on the surface of the anodized group after immersion in SBF for five days compared to the untreated group. The HA phase was significantly higher in all surface-treated groups than in the untreated group, and its precipitation was promoted, depending on the immersion time. The additional manuka coating on the nanotubular layer was predicted to inhibit the improved bioactivity of the TiO_2_ layer because it showed hydrophobicity and high antibacterial activity but was effective in forming a thick HA layer with a high Ca/P ratio. Some authors have confirmed that a hydrophobic and antimicrobial material coating on the surface of the oxide layer can effectively act on the adhesion of compounds in the SBF [26,27]. Moreover, it was reported that some proteins could be adsorbed more strongly on hydrophobic surfaces than on hydrophilic surfaces [28].

However, it was found that the uniformity of HA precipitation on the surface was lower in the group treated with manuka oil with concentrations of 1% or more. This may be related to the morphology of the surface after manuka coating. With manuka coating, it seemed that the surface was dried as the oil was agglomerated by the evaporation of ethanol in the coating diluent. The size and position of the agglomeration increased with increasing concentration (especially at high concentrations (1% and 2%)). The oil-agglomerated position partially impeded the formation of bioactive compounds on the surface of the SBF and prevented uniform HA precipitation. Therefore, it should be remembered that manuka coating can enhance bioactivity, but excessively high concentrations of manuka coating may cause non-uniform precipitation of bioactive compounds.

Many researchers emphasize the importance of retaining cytocompatibility by reducing the concentration of antibacterial drugs because some antibacterial drugs not only inhibit the growth of bacteria but also delay the growth of normal cells [29,30]. Considering this point, the cytotoxicity of osteoblasts with only the oil concentration was evaluated by diluting the culture medium to calculate the actual amount of manuka oil existing on the surface of each manuka-coated group.

As expected, in this study, the proliferation and differentiation capacity of cells was significantly inhibited by the amount of manuka oil in the culture medium. According to ISO 10993-5:2009 [31], manuka oil has cytotoxic potential if the viability of cells is reduced to < 70% of the negative control group by performing an MTT assay. Following this, it was shown that the amount of oil that exists on the surface of groups with a manuka coating with a concentration greater than 1% might have a cytotoxic potential. In the morphological analysis of cells, inhibition of cell growth was observed, as well as a decrease in the number of cells, and the cells grew narrow and long. This means that the cells in medium containing a high concentration of manuka oil experienced more stress compared to normal cells in the control group. Only in the amount that existed on the surface of the group coated with 0.1% manuka oil did the mineralization of mature osteoblasts show no statistically significant difference compared to the control group. However, mineralization in the remaining groups was gradually delayed according to the amount of manuka oil. In particular, the group containing 2% manuka oil showed a reduced mineralization of over 50% compared to the control group.

The results of the previous surface characterization analysis and SBF immersion test demonstrated that the wettability and bioactivity of the surface were improved by the pretreated TiO_2_ layer when manuka coating was performed after anodization on the surface of Ti. The excellent cytocompatibility of TiO_2_ has already been confirmed in published papers [32,33]; therefore, it has been predicted that coating TiO_2_ nanotubes with manuka oil will result in a positive effect on of cell activity. As a result, manuka coating on the anodized Ti surface significantly improved cell proliferation and differentiation ability compared to only manuka oil diluents. In particular, as all manuka-coated groups showed more than 85% better cell viability compared the control group, and no significant cytotoxicity was observed. Unlike in the test using only manuka oil diluents, all anodized and manuka-coated Ti groups showed better cell morphology and adhesion, regardless of coating concentration, leading to excellent cell–cell interactions. Therefore, it was confirmed that the method of applying manuka oil coating after TiO_2_ nanotube layer formation has an excellent effect on cell activity compared to using manuka oil alone.

Even if the use of a high concentration of manuka oil shows a very high antibacterial effect, biosafety in the body should be considered. In this study, 1% and 2% manuka oil coated on surfaces did not show cytotoxicity, while only diluents resulted in a 50% reduction in cell proliferation and differentiation. For this reason, it was judged that a surface-coating concentration of 1% or 2% manuka oil might be cytotoxic in osteoblastic cells. Therefore, except for 1% and 2% of the coating groups, the 0.5% concentration, which showed relatively uniform surface coating and excellent antibacterial properties without inhibiting cell activity, was selected as the test group for the in vivo test. After each surface-treated Ti mesh was placed on the bone defect formed in the femur of the rabbit, bone regeneration was evaluated for 4 and 6 weeks.

Immature bone growth was observed in the control group, which formed only defects without mesh implantation, and complete bone regeneration could not be induced. In addition, unstable and excessive calluses were intensively formed outside the bone defect, causing malunion of the defected area and slight morphological deformation. In GBR using Ti mesh, secondary surgery for implant removal is unavoidable, which often causes difficulties in surgery, depending on the increase in the position and amount of callus enveloped over the mesh [34]. Implantation of the Ti mesh clearly played a role in guiding the bone regeneration of the defect. However, bone regeneration in the case of the mesh without any surface treatment enveloped the mesh, as in the control group. The newly formed bone in this group was cancellous and had a low BMD.

As the TiO_2_-anodized surface has a high affinity for osteoblasts and the ability to rapidly generate bioactive substances in the body [35], it accelerated the concentration of osteocytes around the mesh for a short time. However, this also allowed dense bone to be regenerated outside the bone defect.

On the other hand, the manuka-coated TiO_2_ nanotubular surface showed the highest BMD and BV among the test groups, as compared to bone formation inside the defect of each group, and had the effect of preventing the growth of new bone passing through the mesh. It has been reported that a super-hydrophobic surface can increase the differentiation of osteoblasts and be effective for fast bone bonding [36,37]. Therefore, the hydrophobic surface with the manuka coating led to excellent bone regeneration by effectively acting on bone-cell differentiation and intercellular interaction, as well as maintaining the excellent biocompatibility improved by the TiO_2_ layer.

This study demonstrated the antibacterial effect and biosafety of manuka oil. In addition, it is predicted that the antibacterial manuka coating on the TiO_2_ nanotubular surface can provide biofunctionality in Ti implants and enhance cytocompatibility and stable bone healing. Although these results have sufficient advantages as a multifunctional coating technique for biomaterial for in vitro and vivo tests, selective acceptance by users is very important.

In particular, the optimal concentration of manuka oil was determined by controlling the total amount of manuka, depending on the implant’s surface area. Therefore, for applications in the clinical field, the amount of manuka oil should be recalculated, considering the change in surface area according to the type of implant. In this step, there is a concern that biosafety may decrease when excessively coated manuka oil increases in area. In addition, for further preclinical tests and clinical applications, an adequate choice of the sample size of the animal groups is very important to ensure the reliability of the results. The results presented in this study were used to perform statistical analysis of BV and BMD relative to bone regeneration based on n=4 per animal group. This result is already significant, though even more significant results could be obtained with *n* = 6 per animal group [38]. Additional research encompassing a study of the optimal number of animals per group is needed in the view of possible future clinical applications.

## 5. Conclusions

The manuka oil used in this study, obtained from the East Cape region in New Zealand, was rich in the β-triketone chemotype leptospermone. This chemical compound showed strong inhibitory effects against several pathogenic bacteria, including representative oral bacteria, even at very low oil concentrations.

In this study, to increase the leptospermone-loading capacity on Ti mesh and to provide biofunctionality, the TiO_2_ nanotubular layer was anodized on the surface of the Ti mesh. This pretreated layer effectively enhanced surface hydrophilicity and promoted hydroxyapatite precipitation in the SBF and fast initial bone regeneration. In addition, the nanotubes on the surface of Ti were able to effectively load manuka oil and improve cytocompatibility in manuka oil diluent. Among various concentrations, it was determined that using manuka oil in the concentration range of 0.02% to less than 1% can have a synergistic effect on antibacterial activity and result in excellent biocompatibility. In particular, the group coated with 0.5% manuka oil after anodization promoted bone formation only inside the femoral bone defect, without callus formation outside the Ti mesh.

In conclusion, the hydrophobicity, antibacterial properties, and high bioactivity of manuka oil coating a TiO_2_ nanotube layer can be expected not only to prevent inflammation and stenosis of the connective tissue around the mesh but also to be effective in stable and rapid bone regeneration. Thus, it is thought that this coating method could be valuable in mesh for GBR of bone defects because the new bone from the surface of the mesh can be more easily and effectively separated during secondary surgery for implant removal after complete bone regeneration.

## Figures and Tables

**Table 1:** Conditions of experimental groups.

Name	Sample Condition
T	(T) Pure Titanium
TN	(T) Pure Titanium + (N) Heat-treated Nanotubes
TN0.1M	(T) Pure Titanium + (N) Heat-treated Nanotubes + 0.1% Manuka oil
TN0.5M	(T) Pure Titanium + (N) Heat-treated Nanotubes + 0.5% Manuka oil
TN1M	(T) Pure Titanium + (N) Heat-treated Nanotubes + 1.0% Manuka oil
TN2M	(T) Pure Titanium + (N) Heat-treated Nanotubes + 2.0% Manuka oil

**Table 2:** Determination of MIC (minimum inhibition concentration) and MBC (minimum bactericidal concentration) of manuka oil against the five bacterial species, and the diameter changes of inhibition-zone in disc diffusion test according to increase of concentration.

<Manuka Oil>	MIC	MBC	Inhibition Zone Diameter (mm)											
Concentration (%)			20.00	10.00	5.00	2.50	1.25	0.63	0.31	0.16	0.08	0.04	0.02	0.00
*Streptococcus mutans*	0.08	2.5	12.4	10.4	9.2	8.7	6.5	2.2	1.3	-	-	-	-	-
*Streptococcus anginosus*	0.08	0.31	12.3	11.2	10.7	9.9	8.8	7.5	5.1	4.2	1.4	-	-	-
*Staphylococcus epidermidis*	0.02	0.31	10.9	9.2	8.2	7.4	6.0	4.2	3.1	2.3	1.7	0.6	-	-
*Porphyromonas gingivalis*	0.16	1.25	25<	25<	25<	25<	6.9	5.1	4.8	2.6	1.5	-	-	-
*Escherichia coli*	1.25	10.0	4.2	2.3	1.4	-	-	-	-	-	-	-	-	-

**Table 3:** Compositional changes in the surface after immersion in SBF for five and ten days.

10 Days	T	TN	TN0.1M	TN0.5M	TN1M	TN2M
Ti	38.68	20.43	16.03	15.25	15.41	10.44
O	50.08	59.02	60.53	60.18	60.5	58.71
P	5.18	8.73	9.95	10.32	10.15	12.88
Ca	6.05	11.82	13.48	14.24	13.94	17.97

## Data Availability

Data can be available upon request from the authors.
